# The natural history of primary progressive multiple sclerosis: insights from the German NeuroTransData registry

**DOI:** 10.1186/s12883-023-03273-9

**Published:** 2023-07-05

**Authors:** Stefan Braune, Sandra Bluemich, Carola Bruns, Petra Dirks, Jeanette Hoffmann, Yanic Heer, Erwan Muros-Le Rouzic, Arnfin Bergmann, NTD Study Group, Walter Albrecht, Felix Bischof, Foroogh Bittkau, Simon Bittkau, Kin-Arno Bohr, Bettina Borries, Bernd Brockmeier, Dagmar Brummer, Bernhard Bühler, Wolfgang Butz, Lukas Cepek, Lars Claassen, Jürgen Dee, Lienhard Dieterle, Eckehard Drees, Christoph Engelmann, Michael Ernst, Oliver Fasold, Johannes Fischer, Michael Flach, Robert Fleischer, Lea Friedrich, Anke Friedrich, Michael Fritzinger, Klaus Gehring, Stephanie Gierer, Stephan Gierer, Jens Gößling, Eva Grips, Andreas Hans-Joachim Haldenwanger, Andreas Harth, Rolf Hartmann, Roland Helm, Heinz-Peter Herbst, Christian Hofer, Werner Erwin Hofmann, Alexander Hoge, Sibylla Hummel, Benno Ikenberg, Heike Israel-Willner, Ralf Jankovits, Boris-Alexander Kallmann, Ulrich Kausch, Marc Keppler, Kirn Kessler, Ulrike Kirchhöfer, Jürgen Kirchmeier, Rupert Knoblich, Thomas Knoll, Philipp Knorn, Monika Köchling, Anselm Wolfgang Kornhuber, Bernd Kramer, Michaela Krause, Martin Krauß, Ralf Kubalek, Jürgen Kunz, Harald Landefeld, Thomas Lange, Klaus Lehmann-Horn, Esther Lippert, Karla Lippmann, Walter Maier-Janson, Martin Märkl, Said Masri, Christof Moser, Clemens Neusch, Julius Niemann, Tilmann Paschke, Anna Sybilla Peikert, Andreas Peikert, Henning Peters, Robert Pfister, Gerd Reifschneider, Stefan Ries, Christoph Rieth, Holger Roick, Gerhard Dieter Roth, Roland Roth, Ali Safavi, Joachim Saur, Brigitte Schmitt-Roth, Erich Franz Scholz, Herbert Schreiber, Klaus Schreiber, Christoph Schrey, Carsten Schumann, Martin Seiler, Karl-Otto Sigel, Viola Sikora, Nikolaos Sotiriadis, Stefanie Spiegel, Detlef Städt, Torsten Sühnel, Klaus Tiel-Wilck, Jochen Christoph Ulzheimer, Barbara Sofie Unsorg, Silvia Voith, Achim Stephan Wannenmacher, Hildegund Weber, Markus Weih, Bernd Wendtland, Andreas Wiborg, Martin Wimmer, Thomas Winker, Isaak Wontroba, Monika Wüstenhagen

**Affiliations:** 1 NeuroTransData, Neuburg an der Donau, Germany; 2 Roche Pharma AG, Grenzach-Wyhlen, Germany; 3 F. Hoffmann-La Roche Ltd, Basel, Switzerland; 4 PricewaterhouseCoopers (PwC), Zurich, Switzerland

**Keywords:** Primary progressive multiple sclerosis, Natural history, Retrospective study, Cohort studies, Quality of life

## Abstract

**Background:**

Primary progressive multiple sclerosis (PPMS) is characterised by gradual worsening of disability from symptom onset. Knowledge about the natural course of PPMS remains limited.

**Methods:**

PPMS patients from the German NeuroTransData (NTD) MS registry with data from 56 outpatient practices were employed for retrospective cross-sectional and longitudinal analyses. The cross-sectional analysis included a contemporary PPMS cohort with a documented visit within the last 2 years before index date (1 Jan 2021). The longitudinal analysis included a disease modifying therapy (DMT)-naïve population and focused on the evolution of expanded disability status scale (EDSS) from the first available assessment at or after diagnosis within the NTD registry to index date. Outcome measures were estimated median time from first EDSS assessment to first 24-week confirmed EDSS ≥ 4 and ≥ 7. Besides EDSS change, the proportion of patients on disability pension were described over time.

**Results:**

The cross-sectional analysis included 481 PPMS patients (59.9% female, mean [standard deviation, SD] age 60.5 [11.5] years, mean [SD] EDSS 4.9 [2.1]). Estimated median time from first EDSS assessment after diagnosis to reach 24-week confirmed EDSS ≥ 4 for DMT-naïve patients was 6.9 years. Median time to EDSS ≥ 7 was 9.7 years for 25% of the population. Over a decade mean (SD) EDSS scores increased from 4.6 (2.1) to 5.7 (2.0); the proportion of patients on disability pension increased from 18.9% to 33.3%.

**Conclusions:**

This study provides first insights into the German NTD real-world cohort of PPMS patients. Findings confirm the steadily deteriorating course of PPMS accompanied by increasingly limited quality of life.

**Supplementary Information:**

The online version contains supplementary material available at 10.1186/s12883-023-03273-9.

## Introduction

Multiple sclerosis (MS) is a chronic inflammatory disease of the central nervous system, affecting approximately over 700,000 people in Europe and more than 2.5 million worldwide [1]. Most patients have a relapsing course of MS at onset characterised by relapses and remissions of neurological symptoms, which can be followed by a secondary progressive phase. However, in about 10–15% of patients the disease course is steadily progressing from the start, without or with occasional relapses [2, 3]. While the relapsing–remitting MS (RRMS) disease course shows a female preponderance, females and males are equally affected by primary progressive MS (PPMS) [2]. Median symptom onset in people with PPMS occurs at the age of 40 years, about 10 years later than in RRMS [4]. Clinical symptoms at onset of PPMS include gait difficulties due to muscle weakness, spasticity, and balance impairments, as well as disturbance in fine motor movements and sphincter control [5]. Unlike RRMS, treating the progressing forms of MS remains a challenge with limited options of approved disease modifying therapies (DMTs). Ocrelizumab, a recombinant humanised monoclonal antibody that selectively targets CD20-expressing B cells, is currently the only approved medication for PPMS. Approval was based on results from the randomised, double-blind, placebo-controlled ORATORIO trial [6].

Due to the low proportion of MS patients affected with PPMS, there are few real-world cohorts with sufficiently large numbers of patients to allow a representative investigation of the natural history of PPMS. Relevant data remains limited and heterogeneous on patient characteristics and trajectories in disability over the long-term course of PPMS. Time estimates to reach disability milestones, reflecting a severely limited ability to walk, from symptom onset differ greatly between studies, ranging from 3 to 8 years to reach the Expanded Disability Status Scale (EDSS) 4, for instance [7, 8]. Evaluating predictors of progression remains inconclusive and produced varying results between cohorts [7, 9, 10]. Using data from the German NeuroTransData (NTD) MS registry, we analysed baseline characteristics and disease course of PPMS patients in a real-world setting. We thereby aim to facilitate better understanding of the natural disease course of PPMS.

## Methods

This observational cohort study [SG43202] was based on data from the German NTD MS registry. Data cut-off was 1 Jan 2021.

### German NTD MS registry

The NTD is a Germany-wide network of neurologists and psychiatrists founded in 2008. Currently, the NTD network includes 164 specialists in 56 practices serving about 600,000 outpatients per year. Each practice is certified according to network-specific and ISO 9001 criteria. Compliance with these criteria is audited annually by an external certified audit organisation. The NTD MS registry is a disease specific database digitally capturing demographic, clinical history, and clinical variables from MS patients in a real-world setting. It currently includes about 25,000 patients with MS. All patients gave informed consent for participation and agreed to any secondary use of their data. Standardised clinical assessments of functional system scores and expanded disability status scale (EDSS) calculation are performed by certified raters (http://www.neurostatus.net/). All personnel undergo regular training and both automatic and manually executed queries are implemented to ensure data quality. All data are pseudonymised and pooled to form the MS registry database [11].

The Institute for Medical Information Processing, Biometry and Epidemiology (Institut für medizinische Informationsverarbeitung, Biometrie und Epidemiologie) at the Ludwig Maximilian University in Munich, Germany, manages codes and acts as an external trust centre. Pooled data are stored on NTD servers and other NTD-controlled storage technology. The data acquisition protocol was approved by the ethical committee of the Bavarian Medical Board (Bayerische Landesärztekammer; 14 Jun 2012, No. 11144) and reapproved by the ethical committee of the Medical Board North-Rhine (Ärztekammer Nordrhein, 25 April 2017, ID 2017071). The study conforms to the World Medical Association Declaration of Helsinki as published on the website of the Journal of American Medical Association, the Guidelines for ‘Guidelines for Good Pharmacoepidemiology.

Practices’ GPP published by the International Society of Pharmacoepidemiology (ISPE) and the laws and regulations of Germany.

### Patients

The analyses included PPMS patients diagnosed (for the first time or confirmatory) following common clinical practice and guidelines by a neurologist belonging to the NTD network.

### Cross-sectional analysis

The cross-sectional analysis aimed at describing a contemporary PPMS population with the last visit ≤ 2 years before index date 1 Jan 2021. Patient characteristics were described, including demographics, age at onset and diagnosis of disease, disease duration, EDSS, treatment history with disease modifying therapies (DMTs) and supportive care. Health-related quality of life was assessed by the EQ-5D-5L, a widely used generic measure of health status consisting of a short descriptive system questionnaire and a visual analogue scale (EQ VAS). The descriptive system assesses health in five dimensions (mobility, self-care, usual activities, pain/discomfort, anxiety/depression), each of which has five levels of response (1 = no problems, 2 = slight problems, 3 = moderate problems, 4 = severe problems, 5 = extreme problems/unable to). This questionnaire provides a descriptive profile that can be used to generate a health state profile, whereby 11111 (no problems in any dimension) indicates the best and 55555 (extreme problems in all dimensions) the worst health state. To derive a summary index score an appropriate value set is needed. A health state index score was calculated from individual health profiles using the German Value Set for the EQ-5D-5L as reference. The index score ranges from -0.661 (worst health state) to 1 (best health state) [12]. The EQ VAS assesses how the patient rates their perceived health from 0 (the worst imaginable health) to 100 (the best imaginable health) [13]. Written permission regarding the use of the EQ-5D-5L was obtained from the EuroQol Office.

### Longitudinal analysis

All patients with a PPMS diagnosis and at least two EDSS scores (baseline EDSS < 4 for the time to event analysis to reach EDSS 4; or a baseline EDSS < 7 for the time to event analysis to reach EDSS 7) recorded were included. Patients receiving any DMT were censored in the longitudinal analysis at the time of the first DMT initiation in order to characterise the natural course of PPMS. Time to disability progression milestone of EDSS ≥ 4 (patients are fully ambulatory despite severe disability and able to walk without aid 500 m) and ≥ 7 (essentially restricted to wheelchair), both confirmed after ≥ 24 and ≥ 48 weeks, were investigated by time to event analysis (Kaplan Meier) and Cox proportional-hazards model. Subgroup analyses for the time to EDSS milestones included stratification by age (≤ 55 years versus > 55 years at time of diagnosis). A specific subgroup analysis applying the main inclusion criteria of the pivotal PPMS trial of ocrelizumab (ORATORIO trial) included patients with the following criteria: age 18 to 55 years, baseline EDSS in the NTD MS registry between 3.0 and 6.5, disease duration since onset of symptoms < 15 years if EDSS > 5 at first record in NTD MS registry or disease duration < 10 years if EDSS ≤ 5 at first record in NTD MS registry [6]. Median EDSS for each cohort at the beginning of the time to event analysis was calculated. To further examine the evolution of EDSS over time in DMT-naïve PPMS patients, EDSS scores recorded at successive points in time were plotted per year beginning with the first EDSS assessment recorded in the NTD registry at or after PPMS diagnosis and censoring upon DMT initiation. In case a patient had several EDSS assessments in one year, the arithmetic mean was used to determine the EDSS for that year. In addition, the proportion of patients on self-reported disability pension was evaluated over time. Disability pension can be granted to residents in Germany with reduced working capacity (< 6 h per day), independent of the cause, provided the applicant belonged to the insured group of persons of the statutory pension insurance for at least a period of five years before the onset of reduced working capacity.

### Statistics

No formal sample size was pre-calculated. Data were analysed descriptively using R software version 3.6.0 (R Foundation for Statistical Computing, Vienna, Austria). For the health state index and EQ VAS scores, mean and standard deviation (SD) were calculated. Change in EDSS was represented in box plots of median EDSS per year, starting with the first EDSS visit. Kaplan–Meier estimation was used for time-to-event analyses and displayed as a survival function of event-free disease course. Median time to reach EDSS milestones and interquartile range (Q1; Q3) were reported. The time to EDSS ≥ 4 and ≥ 7 analyses excluded patients who already had EDSS ≥ 4 and ≥ 7 at their first EDSS assessment at or after diagnosis, respectively. For estimation of median time to milestones 4 and 7 generalised gamma functions were fitted to the corresponding survival curves [14]. The suitability of this parametric assumption was assessed by comparing estimates for the time to have events for 25% of the population, e.g. for the event to reach 24-week confirmed EDSS 7 (censored at any DMT). Patients were considered at-risk until the last assessment visit recorded prior to data cut-off or censored at time of death or at start of any DMT, whichever occurred first.

## Results

### Cross-sectional analysis

Out of 23,356 MS patients recorded in the registry at index date, 18,289 (78.3%) were diagnosed with RRMS, 1168 (5.0%) with PPMS, 3240 (13.9%) with SPMS and 659 (2.8%) were not further classified. Of 1168 PPMS patients, 481 patients had a documented visit within the last 2 years before index date (1 Jan 2021) and were included in the cross-sectional analysis. Patient characteristics are summarised in Table 1. Mean time between onset of MS symptoms and PPMS diagnosis recorded in the NTD network was 10.5 years, whereas mean time from onset of MS symptoms to cut-off date was 17.2 years. Mean time between first symptoms and first MS diagnosis was 3.2 years. Median EDSS was 5.0 (IQR 3.5–6.5; mean 4.9; SD 2.1). Among the EDSS subscores, the ambulatory system was affected most (median 3.0; mean 3.4), followed by pyramidal functions (median 3.0; mean 2.8) (Table 1). Before PPMS diagnosis was confirmed, 12.9% of patients had a prior RRMS diagnosis. At index date, 16.4% of patients had previously received off-label treatment with at least one DMT and 18.5% of patients were on treatment with an approved therapy. Mean (SD) EQ-5D-5L index score was 0.6 (0.3) and mean (SD) EQ VAS was 56.9 (22.3). The results of the categorical responses for the five EQ-5D-5L dimensions are summarised in Table 2. The dimensions mostly affected by moderate to extreme problems were mobility (38.5%), followed by usual activities (30.8%) and pain/discomfort (28.4%). Patients reported lower levels of problems in the dimensions self-care and anxiety/depression. A total of 12.1% and 9.1% of patients received pain medications or antidepressants, respectively (Table 1).

**Table 1:** Baseline characteristics (at index date^a^)

Parameter	*N* = 481
Females, n (%)	288 (59.9)
Age, mean (SD), years	
at index date^a^	60.5 (11.5)
at onset of disease (first symptoms)	43.3 (11.6)
at onset of disease (first MS diagnosis)	46.5 (11.4)
at PPMS diagnosis (confirmed^b^)	53.8 (10.8)
Disease duration, mean (SD), years	
since first MS symptoms	17.2 (10.8)
since PPMS diagnosis (confirmed^b^)	6.7 (4.9)
Time between confirmed diagnosis and EDSS assessment within the registry	0.1 (3.6)
Prior RRMS diagnosis, n (%)	62 (12.9)
Prior SPMS diagnosis, n (%)	7 (1.5)
EDSS, median (IQR); mean (SD)^c^	
Total score	5.0 (3.5–6.5); 4.9 (2.1)
Pyramidal functions	3.0 (2.0–4.0); 2.8 (1.5)
Cerebellar functions	1.0 (0.0–2.0); 1.4 (1.3)
Brainstem functions	0.0 (0.0–1.0); 0.6 (0.9)
Sensory functions	1.0 (0.0–2.0); 1.3 (1.3)
Sphincteric functions	1.0 (0.0–2.0); 1.2 (1.2)
Visual functions	0.0 (0.0–0.0); 0.3 (0.7)
Cerebral functions	1.0 (0.0–2.0); 1.1 (1.1)
Ambulation score	3.0 (0.0–6.0); 3.4 (3.2)
Time from last EDSS assessment to index date^a^, mean (SD), months	9.0 (6.8)
Current DMT Use, n (%)	
Approved DMT	89 (18.5)
Other DMT	41 (8.5)
Untreated	351 (73.0)
Use of pain medication, n (%)	58 (12.1)
Use of antidepressants, n (%)^d^	44 (9.1)
Non-medical support, n (%)	
Physiotherapy	273 (56.8)
Support from family	73 (15.2)
Outpatient care	22 (4.6)
Domestic help	139 (28.9)
Short-term care	1 (0.2)
Daycare	1 (0.2)
EQ5D index value, mean (SD)	0.6 (0.3)
EQ VAS, mean (SD)	56.9 (22.3)
Time from last PRO to index date^a^, mean (SD), months	8.6 (6.5)

*DMT* disease modifying treatment, *EDSS* Expanded Disability Status Scale, *IQR* Interquartile range, *MS* Multiple sclerosis, *PPMS* Primary progressive multiple sclerosis, *PRO* Patient-reported outcome, *RRMS* Relapsing–remitting multiple sclerosis, *SD* Standard deviation, *SPMS* secondary progressive multiple sclerosis, *VAS* visual analogue scale

^a^Index date: 1 Jan 2021

^b^confirmed within the NTD registry

^c^Last assessment, within 2 years from index date

^d^selective serotonin reuptake inhibitors, tricyclic antidepressants, serotonin-norepinephrine reuptake inhibitors, mirtazapine, escitalopram, venlafaxine, noradrenergic and specific serotonergic antidepressants, amitriptyline, neuroleptics, sertraline

**Table 2:** Summary statistics including numbers of patients and proportions of categorical responses for the five EQ-5D dimensions

	Mobility n (%)	Self-care n (%)	Usual Activities n (%)	Pain/discomfort n (%)	Anxiety/depression n (%)
Level 1 (No problems)	38 (7.9)	110 (22.9)	54 (11.2)	55 (11.4)	107 (22.2)
Level 2 (Slight problems)	42 (8.7)	57 (11.9)	64 (13.3)	73 (15.2)	83 (17.3)
Level 3 (Moderate problems)	65 (13.5)	49 (10.2)	85 (17.7)	94 (19.5)	53 (11.0)
Level 4 (Severe problems)	85 (17.7)	34 (7.1)	45 (9.4)	40 (8.3)	18 (3.7)
Level 5 (Extreme problems/unable to do)	35 (7.3)	16 (3.3)	18 (3.7)	3 (0.6)	3 (0.6)
Missing	216 (44.9)	215 (44.7)	215 (44.7)	216 (44.9)	217 (45.1)
Total	481 (100)	481 (100)	481 (100)	481 (100)	481 (100)

### Longitudinal analysis

First recorded EDSS visits occurred between 1989 and 2020. Mean time (SD) between PPMS diagnosis recorded in the NTD network and EDSS assessment within the registry was 0.12 years (3.64; *n* = 476).

**Fig. 1:**
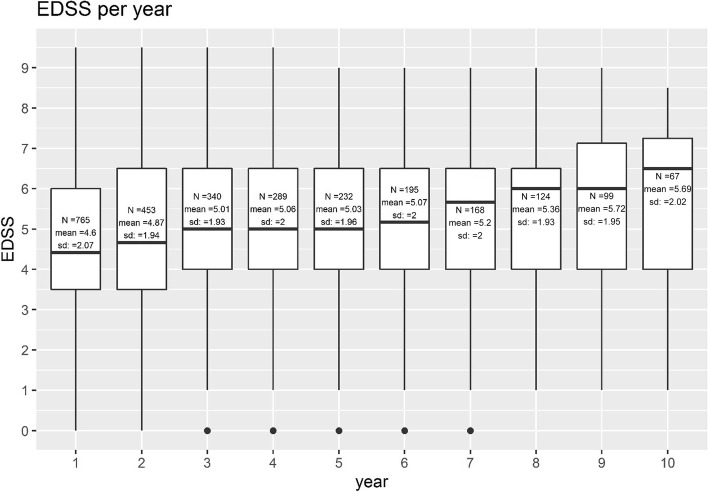
Box plot showing median EDSS of DMT-naïve PPMS patients per year, starting with the first EDSS visit

**Fig. 2:**
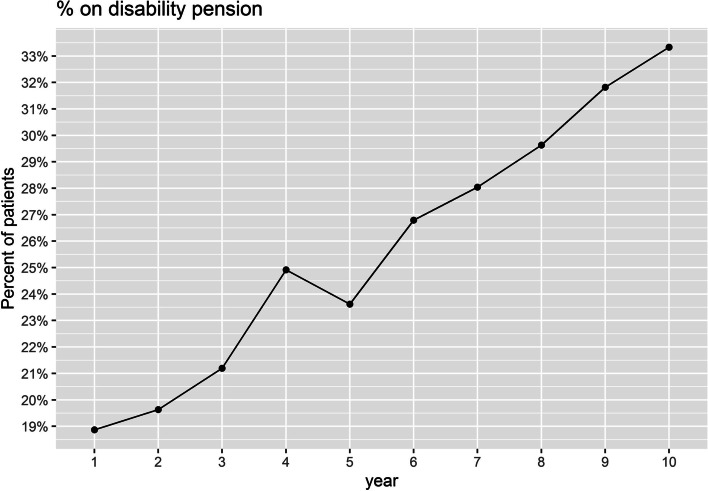
Proportion of PPMS patients without DMT on disability pension over time relative to the index 01.01.2021 (index date)

The course of EDSS over 10 years was marked by a deteriorating tendency. Mean EDSS at diagnosis or first recorded EDSS in the registry was 4.6 (SD 2.1; median 4.42; IQR 3.5–6.0; *n* = 765) and it gradually increased to 5.7 (SD 2.0; median 6.5; IQR 4.0–7.3; *n* = 67) within 10 years (Fig. 1). The proportion of patients on self-reported disability pension increased over time from 18.9% in year 1 to 33.3% in year 10 (Fig. 2).

**Fig. 3:**
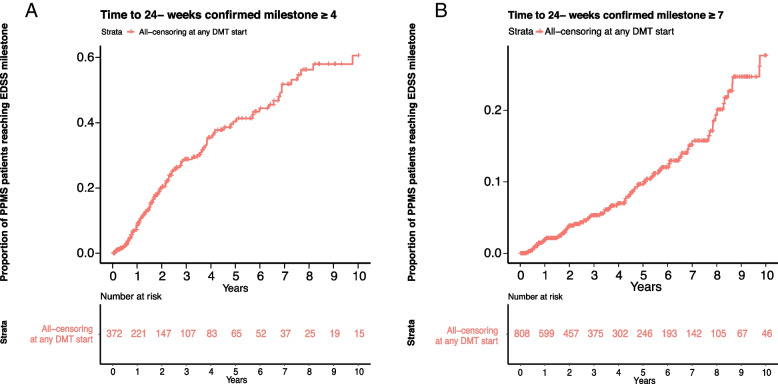
Time from first EDSS assessment at or after diagnosis to 24-week confirmed EDSS score of ≥ 4 (**A**) and ≥ 7 (**B**) Kaplan–Meier survival curve for all DMT-naïve PPMS patients. Patients were censored at start of any DMT. Patients were considered at-risk until the last assessment visit recorded prior to data cut-off or censored at time of death or at start of any DMT whichever occurred first

A total of 372 patients met the inclusion for the time to EDSS ≥ 4 analysis with a baseline median EDSS 3.0. Estimated median time to reach 24-week confirmed EDSS ≥ 4 was 6.9 years (Q1: 2.4; Q3: not reached) (Fig. 3A). A total of 808 patients met the inclusion for the time to EDSS ≥ 7 analysis with a baseline median EDSS 4.0. The extrapolated median time to EDSS ≥ 7 was 16.3 years, however, only a small number of EDSS ≥ 7 events occurred (*n* = 69). The estimated time for 25% of the population to reach EDSS ≥ 7 was 9.7 years (Fig. 3B). Time-to-event plots for 48-week confirmed EDSS milestones of ≥ 4 and ≥ 7 showed a similar course (Supplementary Figures S1 and S2).

**Fig. 4:**
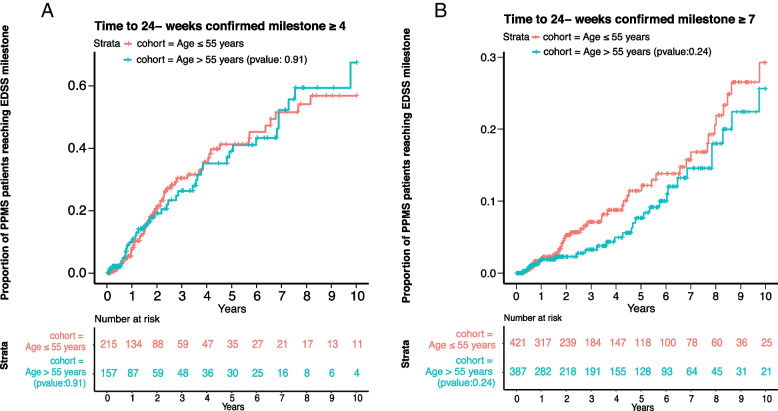
Time from first EDSS assessment at or after diagnosis to 24-week confirmed EDSS score of ≥ 4 (**A**) and ≥ 7 (**B**) Kaplan–Meier survival curve for subgroups of all DMT-naïve PPMS patients ≤ 55 and > 55 years at the time of PPMS diagnosis. Patients were censored at start of any DMT. Patients were considered at-risk until the last assessment visit recorded prior to data cut-off or censored at time of death or at start of any DMT whichever occurred first

Subgroup analyses of patients aged ≤ 55 years and > 55 years at time of diagnosis indicated no significant difference in the time to reach EDSS ≥ 4 (*p* = 0.91) and ≥ 7 (*p* = 0.24) (Fig. 4A and B). Median time to reach EDSS ≥ 4 was 6.8 (Q1: 2.3; Q3: not reached) and 6.9 years (Q1: 2.9; Q3: not reached) for patients ≤ 55 and > 55 years, respectively. Median time to EDSS ≥ 7 was not calculated due to the small number of events (*n* = 41 and *n* = 28, respectively). Estimated time for 25% of the population to reach EDSS ≥ 7 was 8.6 in patients ≤ 55 years and 9.7 years in patients > 55 years at time of diagnosis. Corresponding analyses were similar for 48-week confirmed EDSS milestones ≥ 4 and ≥ 7 (Supplementary Figures S3 and S4).

A subgroup of 84 or 191 patients also fulfilled the criteria corresponding to the inclusion criteria of the ORATORIO pivotal trial and were examined regarding time to EDSS ≥ 4 and ≥ 7. In this cohort, the median time to reach EDSS ≥ 4 was 2.6 years (Q1: 1.3; Q3: not reached). Median time to EDSS ≥ 7 was not reached due to the small number of events (*n* = 7). Corresponding analyses were consistent for 48-week confirmed EDSS ≥ 4 and ≥ 7.

## Discussion

This study provides first insights into the German NTD real-world cohort of PPMS outpatients to describe and thus, better understand the current PPMS population as well as the natural disease course of PPMS based on a national registry.

The NTD registry includes a large MS cohort with about 78, 14 and 5% of MS patients diagnosed with RRMS, SPMS and PPMS, respectively, which is in line with the distribution of MS phenotypes reported in Germany whereas higher numbers have been reported internationally [15–18]. As of January 2021, PPMS patients had a mean age of 60.5 years, median EDSS was 5.0 (mean 4.9 (2.1)) and about 60% were female. Clinical characteristics of the underlying study cohort such as mean age at disease onset (43.3 years) and limited walking ability as a predominant MS related symptom were representative of the PPMS population [4, 5]. The reported time delay of 3 years between presentation of first symptoms and MS diagnosis (approx. 13% with the initial diagnosis of RRMS) not only reflects the well-known challenges in a confirmed PPMS diagnosis [19], but also indicates an initial disease course with only slow progression in the present population [20]. Nevertheless, health related quality of life assessed by the EQ-5D-5L was lower (index 0.6, EQ VAS 56.9) than in the German general elderly population over 65 years of age (index 0.8, EQ VAS 73.2) [21]. Over a maximum observation period of 14 years after first diagnosis, patients have reached a high level of disability and 25% of patients reported severe to extreme problems with mobility.

The disease modifying treatment of PPMS remains a major challenge; therapeutic options are limited with only one approved DMT to date. While almost two thirds of PPMS patients enrolled in the NTD registry were without any DMT, 18.5% received the approved medication ocrelizumab. This seemingly high number of untreated patients might be due to the increased mean age of the cohort at confirmed diagnosis within the registry (53.8). Although the European summary of product characteristics does not prescribe an age limit for the use of ocrelizumab in early PPMS, the pivotal trials only included patients up to the age of 55 years and the interdisciplinary network “Kompetenznetz Multiple Sklerose” (KKNMS) recommends a careful benefit-risk assessment before initiating treatment with ocrelizumab especially for patients with older age [22]. Additionally, the therapeutic indication of ocrelizumab in early PPMS requires MRI evidence of inflammatory activity which assessment is not standardised in clinical practice. Therefore, it is challenging to obtain consistent data on MRI activity needed for treatment decisions. Reported off-label use of immunomodulatory treatments in 8.5% of patients again highlights the unmet medical need and has also been described in a high proportion of PPMS patients enrolled in the German ocrelizumab compassionate use program [23, 24]. Symptomatic therapies represent an essential component of the medical care for patients with PPMS. In line with data from the German DMSG registry, pain and depression were among the most common concomitant symptoms treated with medications [25]. Furthermore, a large proportion of the NTD population is dependent on non-medical support such as physiotherapy and domestic help. Informal care gains in importance as well with an increasing degree of disability [26]. Although a Europe-wide data collection in MS patients indicates that 46% of patients with MS receive support from family members [27] this has only been reported by 15.2% of PPMS patients in the NTD registry.

With the growing efforts to provide PPMS patients with improved or new treatment options [28, 29], a better understanding of the natural disease course is needed to be informed about long-term trajectories and potential therapeutic effects. In the present study the time to reach major EDSS milestones (EDSS 4 and 7), which are associated with a severe impairment of mobility, have been analysed. The time from initial or confirmed PPMS diagnosis in the physician network of NTD to EDSS ≥ 4 was about 7 years. Considering the time span of about 10 years between symptom onset and confirmed diagnosis, disease progression was less rapid than in comparable Canadian and European populations in which a time between 3 and 8 years was described from first symptom to reach EDSS ≥ 4 [7, 8, 30]. However, 20% of the patients have an EDSS of ≥ 4 at their first visit, which could imply a bias towards a population with a slowly progressing disease course in this analysis. Reaching EDSS ≥ 7 and the accompanying need for a wheelchair represented a rare event in the overall analysed cohort, only very few patients (*n* = 69) reached this disability status and the extrapolation of data resulted in an estimated time span of about 16 years from confirmed PPMS diagnosis to EDSS ≥ 7. To gain more robust data, the lower quartile was assessed, which was 9.7 years from confirmed diagnosis for 25% of the population to reach EDSS ≥ 7. Again the increase in disability over time until wheelchair use suggests a slower disease progression in the NTD population than described in other cohorts [7, 20]. Although previous studies reported age at disease onset as a prognostic factor for long-term disability in PPMS [8, 10, 30, 31], age at diagnosis did not influence progression in the German NTD cohort.

Whether the disease course of a real-world cohort differs from a selected clinical trial population was investigated using a subgroup fulfilling the main inclusion criteria of the ocrelizumab pivotal trial in PPMS (ORATORIO) [6]. With a median time of 2.6 years from confirmed diagnosis to reach EDSS ≥ 4 versus 7 years in the total population this subgroup seems to be affected by a more severe disease course. However, due to low patient numbers fulfilling the respective criteria and a short follow-up, these results should be interpreted cautiously.

The consideration of mean EDSS change over a 10-year follow-up showed a moderate but clinically meaningful increase of about 1 EDSS point from 4.6 to 5.7, which appears noticeably lower than the predicted average EDSS increase per year of 0.24 calculated on the basis of pooled individual case data from two German databases [32]. Despite the less pronounced increase in disability measured by the EDSS, the proportion of patients on disability pension increased steadily from 18.9% to 33.3%. This is considerably higher than the proportion of 2% in the German general population [33, 34] and exceeds the proportion reported for MS patients in the German MS registry (22.6%) [25]. However, it should be noted that data from the German MS registry reflects early retirement in MS patients of all phenotypes and not PPMS exclusively. Thus, the higher degree of disability, the older age as well as the progressive phenotype of MS in the analysed population (three factors that are associated with reduced work capacity) must be taken into account when interpreting the data [15, 25, 35].

Due to the real world nature of the NTD MS registry and the secondary data use approach in our analysis, several limitations to the results and their interpretation should be considered. Despite inclusion of outpatients across Germany at all stages of the disease, the patient population from the NTD registry can be prone to selection bias. As these data are captured from outpatient care neurological services only, generalizability of our study findings might be limited. Still, comparison to other German and international data, the mean EDSS of our cohort does not differ substantially. As of Jan 2021, the median EDSS score was 5.0, the baseline (first) mean EDSS for the longitudinal analysis was 4.6. As of September 2022, the German Multiple Sclerosis Society (DMSG) included 2647 PPMS patients from a variety of care facilities with a median EDSS score of 5.5 [36]. Other studies investigating the natural history of PPMS conducted across mainly inpatient MS services different types of MS care centres from the international MSBase registry (24 countries including national MS registries) [20], or in the UK (tertiary referral service) [8] reported baseline median EDSS of 4.0 and 5.8, respectively. Data collection under real-world conditions reflects clinical practice and the observational design of the NTD MS registry with assessments not systematically performed at each visit as well as variability of visit schedules results in incomplete datasets especially in the long-term follow-up. However, a sensitivity analysis between two cohorts with a visit frequency under 150 days vs above 150 days did not reveal any difference. Practical problems inherent to collecting long-term data include patient loss to follow-up, especially for migrating outward from the study area. Consequently, the mean follow-up period of 4.8 years may have been too short to provide robust data on the long-term disease course up to massive physical limitations evident by the need of a wheelchair (EDSS ≥ 7). The precise recording of disease progression in MS patients remains challenging and although the EDSS is considered as the gold standard regarding its assessment, it has well described limitations [37]. Next to a high inter- and intra-rater variability, the EDSS milestones are mainly defined by mobility restrictions ignoring other MS related symptoms that have a strong influence on patient’s quality of life, such as fatigue or impaired upper limb and cognitive functions [37]. Aware of this limitation, additional or composite endpoints are increasingly chosen in clinical trials to allow for a comprehensive evaluation of functional impairments [6, 38–42]. However, a similarly detailed assessment of disease progression is often not feasible in clinical practice.

In summary, this study provides a comprehensive overview on clinical characteristics and the natural history of PPMS patients included in a large contemporary cohort from the German NTD registry. Although the overall population has shown a rather slow progressing course of disease, the data collected underline the significant impact on patients’ everyday life which is reflected in the reduced quality of life and impaired ability to work reported by PPMS patients. There is still a high unmet medical need for disease modifying therapies in progressive MS and despite of limitations the collection of real-world data contributes to a better understanding of long-term disease progression and possible impact of emerging therapies.

## Supplementary Information

**Additional file 1:** **Figure S1.** Time from first EDSS assessment at or after diagnosis to 48-week confirmed EDSS score of ≥4 at or after diagnosis Kaplan-Meier survival curve for DMT-naïve PPMS patients. Patients were censored at start of any DMT. Patients were considered at-risk until the last assessment visit recorded prior to data cut-off or censored at time of death or at start of any DMT whichever occurred first. **Figure S2.** Time from first EDSS assessment at or after diagnosis to 48-week confirmed EDSS score of ≥7 Kaplan-Meier survival curve for DMT-naïve PPMS patients. Patients were censored at start of any DMT. Patients were considered at-risk until the last assessment visit recorded prior to data cut-off or censored at time of death or at start of any DMT whichever occurred first. **Figure S3.** Time from first EDSS assessment at or after diagnosis to 48-week confirmed EDSS score of ≥4 Kaplan-Meier survival curve for subgroups of DMT naïve PPMS patients ≤55 and >55 years at the time of PPMS diagnosis. Patients were censored at start of any DMT. Patients were considered at-risk until the last assessment visit recorded prior to data cut-off or censored at time of death or at start of any DMT whichever occurred first. **Figure S4.** Time from first EDSS assessment at or after diagnosis to 48-week confirmed EDSS score of ≥7 Kaplan-Meier survival curve for subgroups of DMT-naïve PPMS patients ≤55 and >55 years at the time of PPMS diagnosis. Patients were censored at start of any DMT. Patients were considered at-risk until the last assessment visit recorded prior to data cut-off or censored at time of death or at start of any DMT whichever occurred first.

## Data Availability

Any queries regarding data availability can be forwarded to the corresponding author (StB). The trial results will be made available by scientific publication and reported to the funding body.
