# Supplementary material for: The natural history of primary progressive multiple sclerosis: insights from the German NeuroTransData registry
Source: BMC Neurol. 2023 Jul 5;23:258. doi: 10.1186/s12883-023-03273-9 (PMC10320981; doi:10.1186/s12883-023-03273-9)
Supplement: Supplementary file 1 — Additional file 1: Figure S1. Time from first EDSS assessment at or after diagnosis to 48-week confirmed EDSS score of ≥4 at or after diagnosis Kaplan-Meier survival curve for DMT-naïve PPMS patients. Patients were censored at start of any DMT. Patients were considered at-risk until the last assessment visit recorded prior to data cut-off or censored at time of death or at start of any DMT whichever occurred first. Figure S2. Time from first EDSS assessment at or after diagnosis to 48-week confirmed EDSS score of ≥7 Kaplan-Meier survival curve for DMT-naïve PPMS patients. Patients were censored at start of any DMT. Patients were considered at-risk until the last assessment visit recorded prior to data cut-off or censored at time of death or at start of any DMT whichever occurred first. Figure S3. Time from first EDSS assessment at or after diagnosis to 48-week confirmed EDSS score of ≥4 Kaplan-Meier survival curve for subgroups of DMT naïve PPMS patients ≤55 and >55 years at the time of PPMS diagnosis. Patients were censored at start of any DMT. Patients were considered at-risk until the last assessment visit recorded prior to data cut-off or censored at time of death or at start of any DMT whichever occurred first. Figure S4. Time from first EDSS assessment at or after diagnosis to 48-week confirmed EDSS score of ≥7 Kaplan-Meier survival curve for subgroups of DMT-naïve PPMS patients ≤55 and >55 years at the time of PPMS diagnosis. Patients were censored at start of any DMT. Patients were considered at-risk until the last assessment visit recorded prior to data cut-off or censored at time of death or at start of any DMT whichever occurred first. [file 12883_2023_3273_MOESM1_ESM.zip › supp_figure1_ESM.pdf]

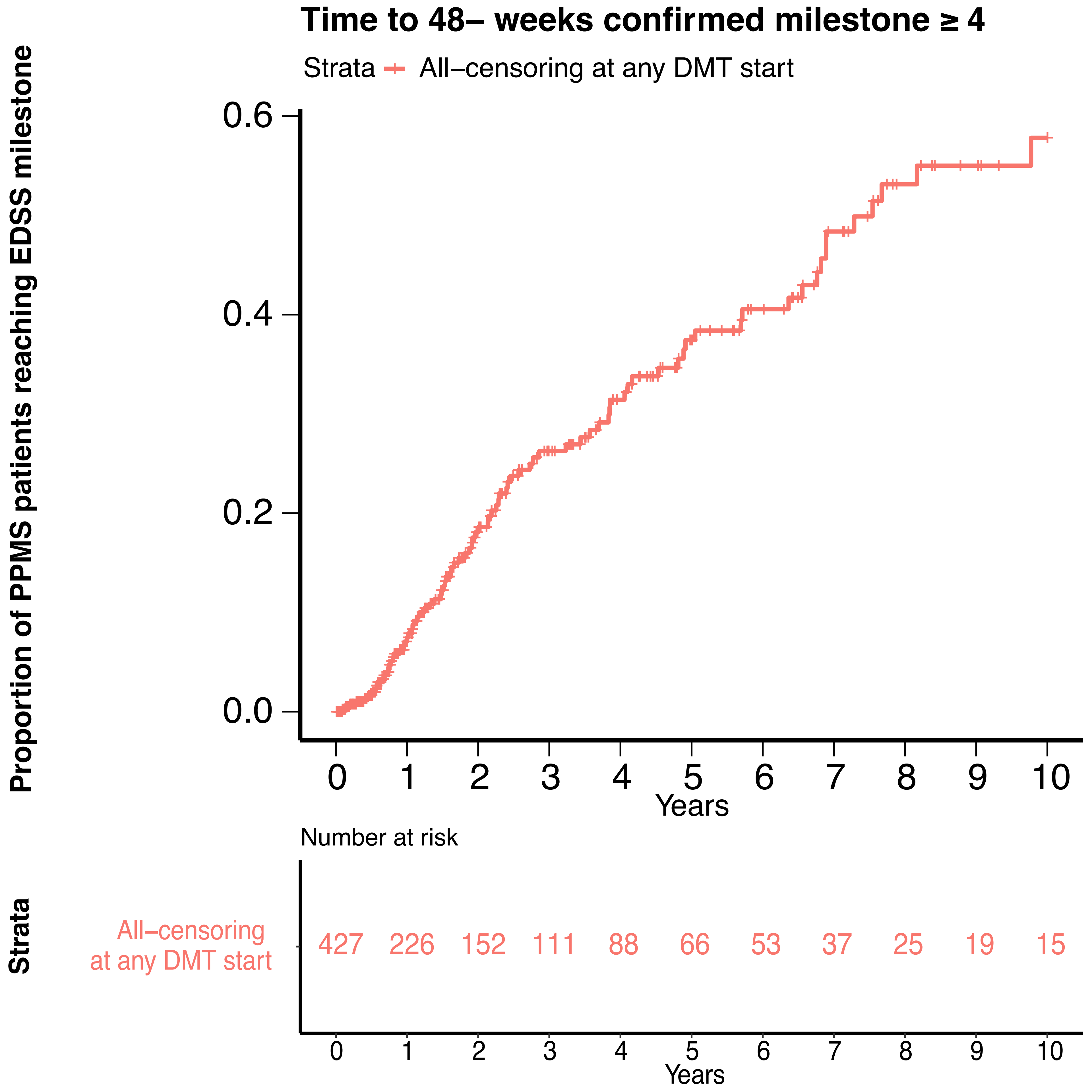

Time to 48– weeks confirmed milestone ≥ 4
Strata
All–censoring at any DMT start
Proportion of PPMS patients reaching EDSS milestone
0.0
0.2
0.4
0.6
0
1
2
3
4
5
6
7
8
9
10
Years
Number at risk
Strata
All–censoring at any DMT start
427
226
152
111
88
66
53
37
25
19
15
Years
