# Supplementary material for: The natural history of primary progressive multiple sclerosis: insights from the German NeuroTransData registry
Source: BMC Neurol. 2023 Jul 5;23:258. doi: 10.1186/s12883-023-03273-9 (PMC10320981; doi:10.1186/s12883-023-03273-9)
Supplement: Supplementary file 1 — Additional file 1: Figure S1. Time from first EDSS assessment at or after diagnosis to 48-week confirmed EDSS score of ≥4 at or after diagnosis Kaplan-Meier survival curve for DMT-naïve PPMS patients. Patients were censored at start of any DMT. Patients were considered at-risk until the last assessment visit recorded prior to data cut-off or censored at time of death or at start of any DMT whichever occurred first. Figure S2. Time from first EDSS assessment at or after diagnosis to 48-week confirmed EDSS score of ≥7 Kaplan-Meier survival curve for DMT-naïve PPMS patients. Patients were censored at start of any DMT. Patients were considered at-risk until the last assessment visit recorded prior to data cut-off or censored at time of death or at start of any DMT whichever occurred first. Figure S3. Time from first EDSS assessment at or after diagnosis to 48-week confirmed EDSS score of ≥4 Kaplan-Meier survival curve for subgroups of DMT naïve PPMS patients ≤55 and >55 years at the time of PPMS diagnosis. Patients were censored at start of any DMT. Patients were considered at-risk until the last assessment visit recorded prior to data cut-off or censored at time of death or at start of any DMT whichever occurred first. Figure S4. Time from first EDSS assessment at or after diagnosis to 48-week confirmed EDSS score of ≥7 Kaplan-Meier survival curve for subgroups of DMT-naïve PPMS patients ≤55 and >55 years at the time of PPMS diagnosis. Patients were censored at start of any DMT. Patients were considered at-risk until the last assessment visit recorded prior to data cut-off or censored at time of death or at start of any DMT whichever occurred first. [file 12883_2023_3273_MOESM1_ESM.zip › supp_figure3_ESM.pdf]

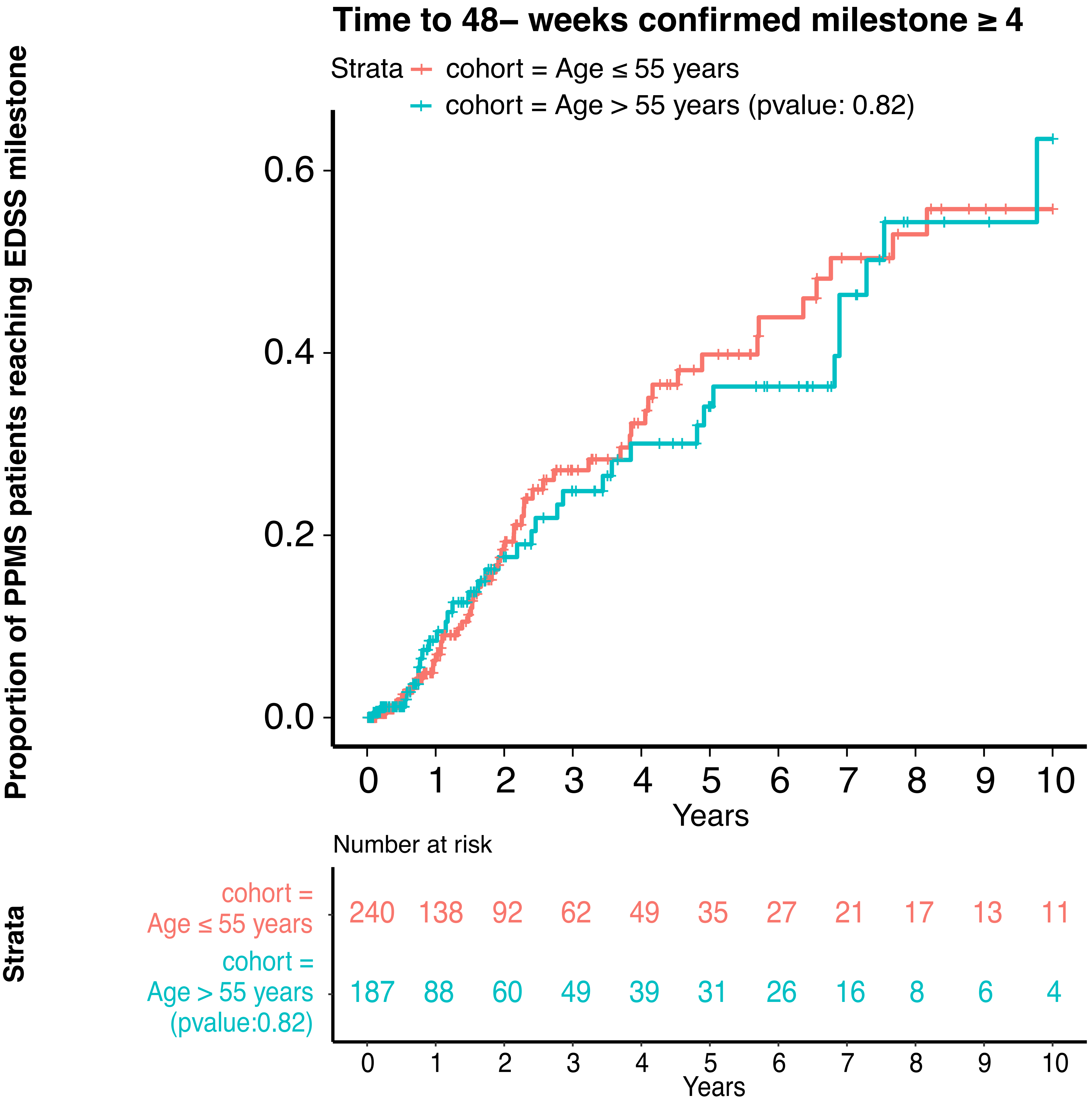
Time to 48– weeks confirmed milestone ≥ 4
Strata
cohort = Age ≤ 55 years
cohort = Age > 55 years (pvalue: 0.82)
Proportion of PPMS patients reaching EDSS milestone
0.0
0.2
0.4
0.6
0
1
2
3
4
5
6
7
8
9
10
Years
Number at risk
Strata
cohort = Age ≤ 55 years
240 138 92 62 49 35 27 21 17 13 11
cohort = Age > 55 years (pvalue:0.82)
187 88 60 49 39 31 26 16 8 6 4
Years
