# Supplementary material for: The natural history of primary progressive multiple sclerosis: insights from the German NeuroTransData registry
Source: BMC Neurol. 2023 Jul 5;23:258. doi: 10.1186/s12883-023-03273-9 (PMC10320981; doi:10.1186/s12883-023-03273-9)
Supplement: Supplementary file 1 — Additional file 1: Figure S1. Time from first EDSS assessment at or after diagnosis to 48-week confirmed EDSS score of ≥4 at or after diagnosis Kaplan-Meier survival curve for DMT-naïve PPMS patients. Patients were censored at start of any DMT. Patients were considered at-risk until the last assessment visit recorded prior to data cut-off or censored at time of death or at start of any DMT whichever occurred first. Figure S2. Time from first EDSS assessment at or after diagnosis to 48-week confirmed EDSS score of ≥7 Kaplan-Meier survival curve for DMT-naïve PPMS patients. Patients were censored at start of any DMT. Patients were considered at-risk until the last assessment visit recorded prior to data cut-off or censored at time of death or at start of any DMT whichever occurred first. Figure S3. Time from first EDSS assessment at or after diagnosis to 48-week confirmed EDSS score of ≥4 Kaplan-Meier survival curve for subgroups of DMT naïve PPMS patients ≤55 and >55 years at the time of PPMS diagnosis. Patients were censored at start of any DMT. Patients were considered at-risk until the last assessment visit recorded prior to data cut-off or censored at time of death or at start of any DMT whichever occurred first. Figure S4. Time from first EDSS assessment at or after diagnosis to 48-week confirmed EDSS score of ≥7 Kaplan-Meier survival curve for subgroups of DMT-naïve PPMS patients ≤55 and >55 years at the time of PPMS diagnosis. Patients were censored at start of any DMT. Patients were considered at-risk until the last assessment visit recorded prior to data cut-off or censored at time of death or at start of any DMT whichever occurred first. [file 12883_2023_3273_MOESM1_ESM.zip › supp_figure4_ESM.pdf]

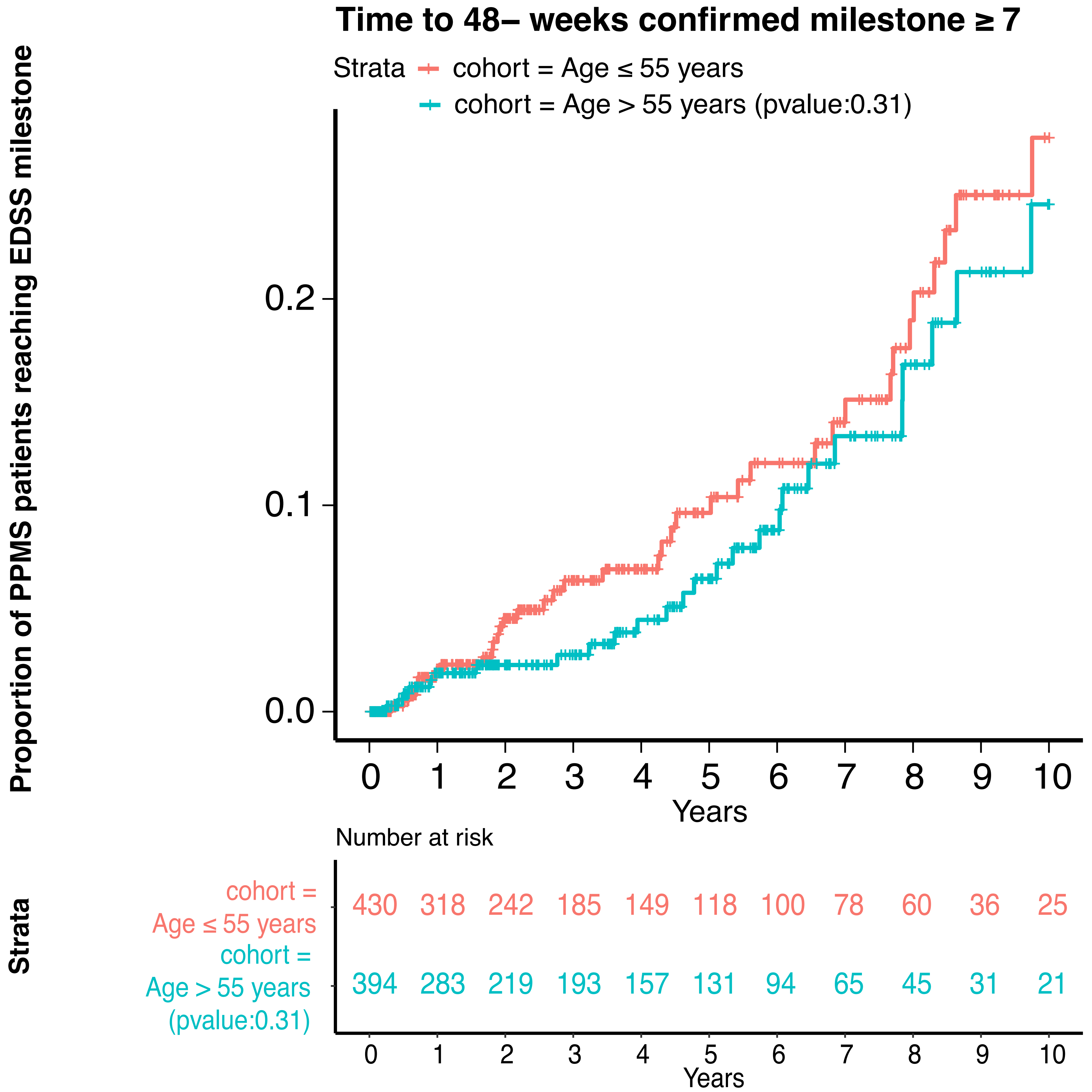

Time to 48– weeks confirmed milestone ≥ 7
Strata
cohort = Age ≤ 55 years
cohort = Age > 55 years (pvalue:0.31)
Proportion of PPMS patients reaching EDSS milestone
0.0
0.1
0.2
0 1 2 3 4 5 6 7 8 9 10
Years
Number at risk
Strata
cohort = Age ≤ 55 years
430 318 242 185 149 118 100 78 60 36 25
cohort = Age > 55 years (pvalue:0.31)
394 283 219 193 157 131 94 65 45 31 21
0 1 2 3 4 5 6 7 8 9 10
Years
